# A Machine Learning Model for the Prediction of No-Reflow Phenomenon in Acute Myocardial Infarction Using the CALLY Index

**DOI:** 10.3390/diagnostics14242813

**Published:** 2024-12-14

**Authors:** Halil Fedai, Gencay Sariisik, Kenan Toprak, Mustafa Beğenç Taşcanov, Muhammet Mucip Efe, Yakup Arğa, Salih Doğanoğulları, Sedat Gez, Recep Demirbağ

**Affiliations:** 1 Department of Cardiology, Harran University Faculty of Medicine, Şanlıurfa 63300, Turkey; kentoprak@hotmail.com (K.T.); drbegenc@gmail.com (M.B.T.); dr.salih63@gmail.com (S.D.); gezsedatt30394@gmail.com (S.G.); rdemirbag@harran.edu.tr (R.D.); 2 Department of Industrial Engineering, Harran University Faculty of Engineering, Şanlıurfa 63300, Turkey; gsariisik@gmail.com; 3 Clinic of Cardiology, Siverek State Hospital, Şanlıurfa 63700, Turkey; muh.efe22@hotmail.com; 4 Clinic of Cardiology, Viranşehir State Hospital, Şanlıurfa 63700, Turkey; yakuparga@hotmail.com

**Keywords:** acute myocardial infarction, no-reflow, STEMI, CALLY index, machine learning, XGBoost, cardiovascular diseases

## Abstract

Background: Acute myocardial infarction (AMI) constitutes a major health problem with high mortality rates worldwide. In patients with ST-segment elevation myocardial infarction (STEMI), no-reflow phenomenon is a condition that adversely affects response to therapy. Previous studies have demonstrated that the CALLY index, calculated using C-reactive protein (CRP), albumin, and lymphocytes, is a reliable indicator of mortality in patients with non-cardiac diseases. The objective of this study is to investigate the potential utility of the CALLY index in detecting no-reflow patients and to determine the predictability of this phenomenon using machine learning (ML) methods. Methods: This study included 1785 STEMI patients admitted to the clinic between January 2020 and June 2024 who underwent percutaneous coronary intervention (PCI). Patients were in no-reflow status, and other clinical data were analyzed. The CALLY index was calculated using data on patients’ inflammatory status. The Extreme Gradient Boosting (XGBoost) ML algorithm was used for no-reflow prediction. Results: No-reflow was detected in a proportion of patients participating in this study. The model obtained with the XGBoost algorithm showed high accuracy rates in predicting no-reflow status. The role of the CALLY index in predicting no-reflow status was clearly demonstrated. Conclusions: The CALLY index has emerged as a valuable tool for predicting no-reflow status in STEMI patients. This study demonstrates how machine learning methods can be effective in clinical applications and paves the way for innovative approaches for the management of no-reflow phenomenon. Future research needs to confirm and extend these findings with larger sample sizes.

## 1. Introduction

Cardiovascular diseases, particularly AMI, remain a leading cause of mortality worldwide. Among AMI patients, those experiencing STEMI face higher mortality rates due to complete vessel occlusion. As per the European Society of Cardiology guidelines, PCI is the standard treatment for STEMI. However, the no-reflow phenomenon, where blood flow cannot be fully restored, exacerbates myocardial infarction by increasing infarct size and is linked to rehospitalization, adverse ventricular remodeling, malignant arrhythmias, heart failure, and higher mortality [1,2,3,4].

Although the exact pathophysiology of no-reflow remains unclear, factors such as hypertension, diabetes, smoking, dyslipidemia, atrial fibrillation, advanced age, delayed presentation, heavy thrombus burden, and repeated balloon dilatation are recognized risk factors [5,6]. Given the uncertainty in pathogenesis and its significant impact on patient outcomes, various risk scores have been developed to predict no-reflow, though their accuracy and ease of use remain debated [7,8,9]. Nevertheless, certain parameters have been identified as predictors of the no-reflow phenomenon, which is associated with elevated mortality and cardiac remodeling. Despite the controversy surrounding these predictors, the studies have demonstrated a correlation between the no-reflow phenomenon and both short- and long-term mortality [8,10,11].

The role of inflammation in no-reflow is of critical importance, and a comprehensive evaluation of this process could prove invaluable in identifying high-risk patients. The CALLY index is an efficacious score that incorporates CRP, albumin, and lymphocytes and indicates the inflammatory, nutritional, and immune status of patients. The CALLY index, which has been linked to prognosis in non-cardiac diseases, may prove useful in predicting no-reflow. An investigation into the concordance of the parameters within the CALLY index may facilitate a more profound understanding of the patient’s inflammatory status. This study examines the potential of the CALLY index as a diagnostic tool for no-reflow patients through a novel mathematical learning approach, with the aim of identifying these high-risk individuals [12,13,14,15].

Despite significant advances, there remains a gap in the literature regarding the predictive accuracy of simple scoring methods for no-reflow in STEMI patients. Few studies have applied interpretable artificial intelligence (AI) techniques to address this issue. ML, a branch of AI that enables data-driven prediction, has seen growing use in healthcare, ranging from genetic disease detection to early cancer diagnosis and medical imaging analysis [16,17]. Furthermore, machine learning has been employed in multicenter retrospective and prospective studies in recent years. It has been demonstrated that this method may prove beneficial in the diagnosis of cardiac and non-cardiac diseases [18,19]. This study will employ ML methods, specifically XGBoost, to classify reflow status and identify the most important risk factors using variable importance scores.

## 2. Materials and Methods

### 2.1. Study Design and Data

**Figure 1:**
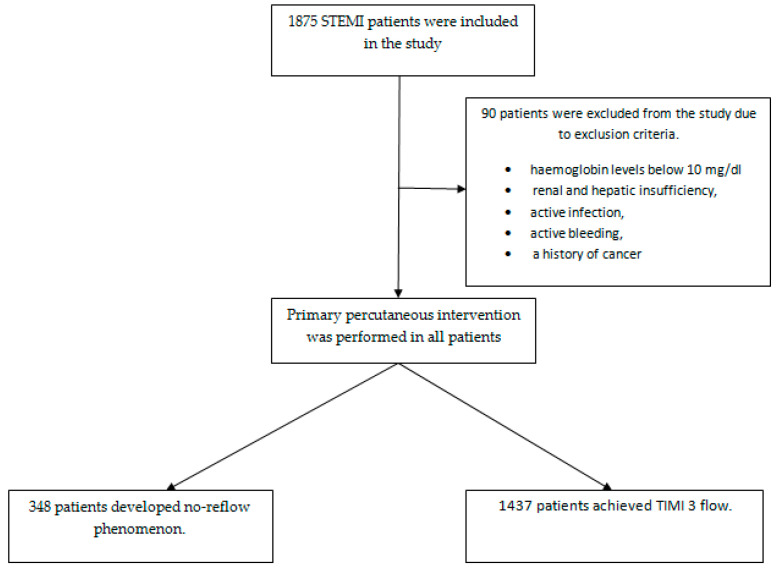
Flowchart of the study.

We retrospectively analyzed 1785 STEMI patients admitted to our clinic between January 2020 and June 2024 who underwent percutaneous coronary intervention. Patients included in this study were carefully selected according to the latest STEMI European Society of Cardiology guidelines [20]. Included patients were consecutively enrolled. All patients were treated with standard antiaggregant and anticoagulant therapy as recommended by ESC guidelines, and primary percutaneous intervention was performed by experienced operators [20]. Patients’ data were accessed through the hospital system, and all angiographic images were reviewed by two experienced cardiologists. Angiographic images were classified into thrombolysis in myocardial infarction (TIMI) blood flow grades. Grade 0 indicates no flow after the point of occlusion. In grade 1, contrast medium flows beyond the occlusion area but fails to opacify the entire artery. Grade 2 indicates opacification of the entire artery distal to the site of occlusion, but at a slower than normal rate, and grade 3 indicates normal coronary flow [21]. According to the TIMI classification, 348 patients who did not achieve TIMI 3 flow were found to have a no-reflow phenomenon. Patients with hemoglobin levels below 10 mg/dl, renal and hepatic insufficiency, active infection, active bleeding, and a history of cancer were excluded from this study. It was assumed that the albumin, lymphocyte, and CRP levels used to calculate the CALLY index would be affected in all these conditions. Changes in these values will only limit the interpretation of the no-reflow phenomenon when analyzing the results. Therefore, patients with these co-morbidities were not included (Figure 1). Ethical approval was duly obtained from the institutional ethics committee for this study, and the study was ensured to comply with the established ethical rules.

### 2.2. Data Collection 

Data pertaining to demographic variables, AMI locations, and comorbidities were extracted from electronic medical records. At the same time, the hematological and biochemical laboratory results from the venous blood taken at the first visit to the emergency department were as follows: urea, creatinine, albumin, CRP, aspartate transaminase, alanine transaminase, troponin, hemoglobin values, hematocrit values, mean platelet volume values, procalcitonin values, platelet counts, and white blood cell, neutrophil, and lymphocyte counts, as well as the neutrophil-to-lymphocyte ratio (NLR) counts. The method of calculating the CALLY index: Albumin × Lymphocyte ÷ (CRP × 10) [22].

### 2.3. Machine Learning–XGBoost

Gradient Boost is a valuable machine learning strategy for solving regression and classification problems, especially in situations where weaker prediction models are often used. In such cases, decision tree ensembles can be effectively used to improve the overall performance of the model. Gradient Boost is a machine learning algorithm that employs a boosting approach to construct a series of relatively weak learners in a sequential manner, subsequently integrating them into a more complex model [23].

XGBoost represents a supervised learning approach that employs gradient boosting machines. Its foundation is based on the principles of gradient boosting and decision tree methods, which are collectively known as “boosting trees”. In comparison to alternative algorithms, it demonstrates a notable advantage in terms of both speed and performance. Furthermore, XGBoost exhibits high predictive capabilities, approximately ten times the speed of other algorithms, and incorporates a number of regularizations that enhance overall performance while reducing overfitting and over-learning. XGBoost can enhance performance by regulating the intricacy of the trees through the utilization of diverse regularization techniques [24].

### 2.4. Machine Learning Modeling and Performance Evaluation

The objective of this study was to identify the most salient features within the dataset that exert a significant influence on the dependent variable. To this end, a variable selection was conducted. The random forest variable selection method was utilized as the method of variable selection. In the present study, the XGBoost algorithm was employed for the purpose of modeling the dataset. The reason for using the XGBoost algorithm in our study is primarily based on its demonstrated superior performance in predicting cardiovascular events, such as the no-reflow phenomenon, as highlighted in the literature. XGBoost is well-known for its ability to deliver high accuracy in classification and regression problems, especially when dealing with imbalanced datasets [25,26]. Additionally, the flexibility it offers in model training and parameter tuning allows for more accurate and reliable results, making it particularly suitable for complex and multidimensional datasets, such as those encountered in cardiovascular data. The dataset was randomly partitioned into an 80:20 training and test set. The n-fold cross-validation approach was utilized in the analysis. In the n-fold cross-validation procedure, the dataset is partitioned into n subsets, and the model is applied to each subset. One of the n components is employed for testing purposes, while the remaining n-1 components are utilized for model training. In this study, the modeling process was conducted using a five-fold cross-validation approach. The evaluation of performance was based on a number of criteria, including accuracy, balanced accuracy, sensitivity, selectivity, positive predictive value, negative predictive value, and F1-score. Moreover, variable importance was calculated, which offers insight into the extent to which input variables influence the output variable.

## 3. Results

### 3.1. Analysis of Clinical and Demographic Data in STEMI Patients

In this study, we analyzed the clinical and demographic data of STEMI patients, focusing on their general health status, risk factors, and laboratory results. Statistical evaluations emphasize the importance of these parameters in the treatment of patients and shed light on which indicators should be taken into account in predicting critical situations such as no-reflow events. Detailed data on these findings are presented below.

Table 1 presents descriptive statistics on clinical and demographic parameters of STEMI patients. The results of the analysis show that the mean age of the patient group was 58.53 (±12.12) years, indicating that the majority of the group consisted of middle-aged and older individuals. The mean body mass index (BMI) was 26.50 (±2.00), indicating the presence of a slightly overweight group. Among the patients included in this study, 35.1% were diagnosed with diabetes mellitus, 66.5% had hypertension, and 62.3% were current smokers. In terms of laboratory results, the mean creatinine was 0.87 mg/dL (±0.28), low-density lipoprotein cholesterol was 108.90 mg/dL (±36.66), and CRP was 1.33 mg/L (±1.57) (Table 1).

Table 2 presents the results of a one-sample t-test to predict the no-reflow event. The analysis includes mean differences, t-values, degrees of freedom (df), *p*-values, and 95% confidence intervals for clinical parameters including age, BMI, glucose, creatinine, low-density lipoprotein cholesterol, CRP, white blood cell count, hematocrit, number of vessels, stent length, SYNTAX score, CALLY index, and NLR.

The results of statistical analysis show that the *p*-values of all parameters are significant (*p* < 0.05). This suggests that all the factors evaluated for predicting the no-reflow event provide a statistically strong basis and that the results are not coincidental. In particular, the mean CALLY index was 3.82 (±8.82), and the high standard deviation of this parameter indicates that there were significant differences between patients. The CALLY index was evaluated as an important indicator for the prediction of the no-reflow phenomenon, with a *p*-value of less than 0.001, emphasizing the need to integrate this parameter into clinical practice.

### 3.2. Analysis and Performance Evaluation of the XGBoost Model

This paper analyzes in detail the output of machine learning models on the CALLY index dataset. The analysis is performed using the Python programming language, and comparisons are made between the actual CALLY index values and the values predicted by various machine learning algorithms.

**Figure 2:**
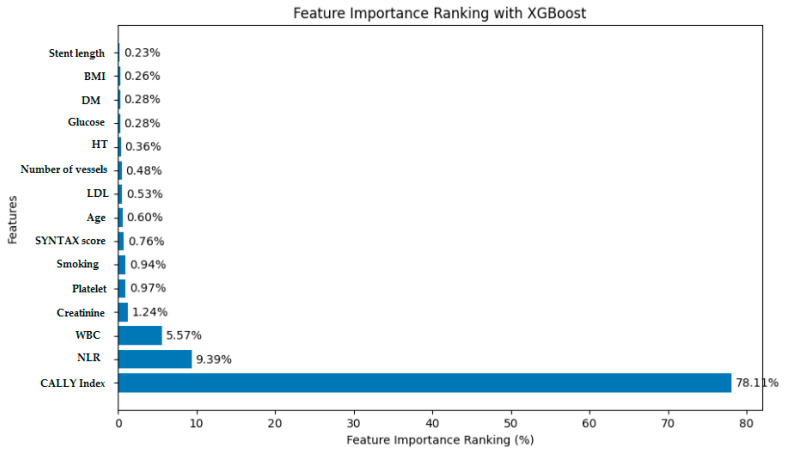
Importance of the CALLY index in the XGBoost model.

Figure 2 examines the output of the XGBoost model and ranks the impact of the CALLY index input features on the model. This analysis provides an important insight into the contribution of each input feature to the prediction results. As a result of the analysis performed with the XGBoost algorithm, it was observed that the CALLY index classification reached a value of 78.11%, higher than the predictions made with the NLR index. This ranking and interaction analysis helps us understand how sensitive the model is to which input features and provides important information for optimizing predictions and increasing model reliability.

A custom prediction model for the CALLY index was developed to evaluate the output of the machine learning model and identify the features that most influence the model predictions. The evaluation process was performed using key metrics such as coefficient of determination (R²), mean absolute error (MAE), and root mean square error (RMSE). For each forecasting model, these metrics were used to objectively assess the performance of the model.

The coefficient of determination (R²) is an important metric used to measure how well the model fits the data; this metric shows how much of the total variance is explained by the model’s predictions. According to the results, the XGBoost algorithm achieved R² = 0.99 in the training set and R² = 0.87 in the test set, indicating that the model fits the data perfectly and works with high accuracy in the test data. Furthermore, the MAE plays an important role in evaluating the model; a low MAE value indicates how close the model’s predictions are to the true values, and hence the prediction performance is high. RMSE measures the overall magnitude of the model’s forecast errors, which allows for a more in-depth analysis of performance. A low RMSE indicates a low error rate in the model’s predictions.

**Figure 3:**
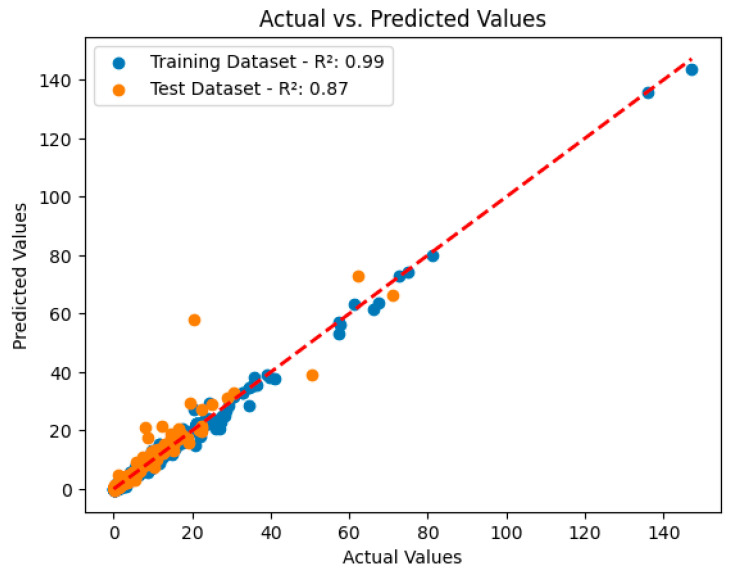
Models predicting the CALLY index values in the XGBoost model.

These metrics are used to determine the success of machine learning models in predicting CALLY index values and to provide important information about the reliability and effectiveness of the research. As shown in Figure 3, the XGBoost algorithm achieved coefficients of determination of R² = 0.99 on the training set and R² = 0.87 on the test set, indicating an overall high success of the model.

**Figure 4:**
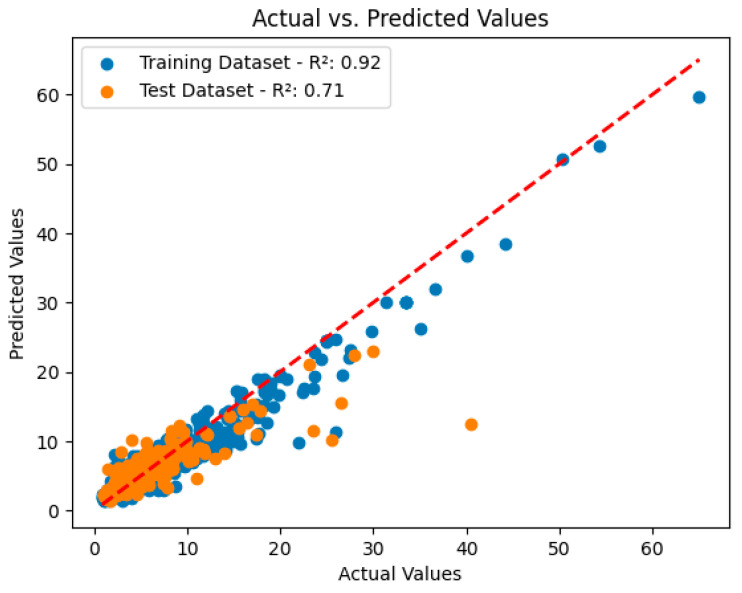
Models predicting the NLR index values in the XGBoost model.

These metrics are used to determine the success of machine learning models in predicting NLR index values and to provide important information about the reliability and effectiveness of the research. According to Figure 4, the XGBoost algorithm achieved coefficients of determination of R² = 0.92 in the training set and R² = 0.71 in the test set. These results indicate that the forecasts for the NLR index are also quite successful but perform less well than the forecasts for the CALLY index. This suggests that the CALLY index may be a more decisive indicator for the prediction of the no-reflow phenomenon. In conclusion, the findings obtained with the XGBoost algorithm reveal the potential of machine learning techniques in the prediction of variables such as the CALLY index and the NLR index. Future studies can investigate the integration of these indices into clinical applications and how they can be used more effectively in patient management.

## 4. Discussion

This retrospective study aimed to evaluate the phenomenon of no-reflow in patients presenting with STEMI who underwent PCI. Specifically, we assessed the utility of the CALLY index, a score based on straightforward biochemical markers—CRP, serum albumin, and lymphocytes—which has previously demonstrated prognostic value in non-cardiac diseases. Furthermore, we employed the XGBoost machine learning algorithm, as opposed to conventional statistical techniques, to assess the predictive power of this score.

Our analysis revealed that the CALLY index demonstrated a high degree of accuracy in identifying patients at risk of the no-reflow phenomenon, with a detection rate of 78.11%. The XGBoost algorithm exhibited strong performance, with coefficients of determination (R²) of 0.99 in the training set and 0.87 in the test set, underscoring the robustness of the model and the reliability of the CALLY index in predicting no-reflow.

Despite the efficacy of rapid and effective PCI, patients presenting with STEMI remain at high risk. The prediction of complications that may develop after STEMI is a key objective in reducing mortality in these patients. To this end, a range of biochemical parameters and imaging modalities have been employed to identify high-risk STEMI patients [10,11,27]. However, the no-reflow phenomenon persists in 5–50% of cases. [28]. Achieving a normal TIMI 3 flow in patients exhibiting the no-reflow phenomenon is not always feasible. It is not always feasible to attain a normal TIMI 3 flow in patients exhibiting no-reflow phenomenon. Achieving TIMI 3 results in the inability to maintain flow and an enlargement of the infarct area. Indeed, numerous studies have demonstrated an elevated risk of mortality and cardiovascular events in patients with no-reflow. The mechanisms believed to be associated with the pathophysiology of no-reflow include the following: distal embolization of plaque and thrombus; microvascular injury; reperfusion myocardial injury caused by radical oxygen production; myocardial necrosis; release of activated tissue factor from dissected plaque; vasoconstriction due to increased alpha-adrenergic tone; and release of thromboxane A2 and serotonin from platelets [29]. It is evident that the underlying cause and the resulting consequence of these intricate mechanisms is a significant elevation in inflammatory processes [30,31,32]. Given the evidence that inflammatory processes are involved in both the initiation and progression of atherosclerosis, the next step was to investigate the relationship between inflammatory state and adverse events in patients with AMI [4]. We also know that inadequate microvascular perfusion, whatever the cause, has been implicated in the physiopathology of the no-reflow phenomenon [4]. This evidence indicates a close relationship between the no-reflow mechanism and myocardial nutrition mechanisms [4]. Consequently, the no-reflow mechanism cannot be attributed to a single causal factor.

The CALLY index is a score comprising three components: CRP, serum albumin, and lymphocytes. Collectively, these elements can be used to assess the level of inflammation, nutritional status, and immune function. Prior research has indicated that the CALLY index is significantly associated with the prognosis and mortality of various diseases, particularly cancers [1]. A review of the literature revealed that the CALLY index has not been extensively studied in the context of cardiac diseases.

The pathophysiology of the no-reflow phenomenon remains poorly understood. In AMI, more myocytes and endothelial cells are lost with increasing ischemic time. The dead cells cause inflammation and interstitial edema, and over time the microvascular bed is disrupted. As ischemic time increases, inflammation increases, microvascular perfusion is impaired, and microvascular perfusion impairment increases ischemic injury and inflammation, creating a vicious cycle [33,34,35]. Inflammation and malnutrition of the myocardial bed are therefore an integral part of the no-reflow phenomenon [30,31,32].

In the present study, we demonstrated that the CALLY index is an effective method for detecting the no-reflow phenomenon in patients with STEMI based on preoperative blood values. Our findings revealed that the XGBoost modeling technique exhibited a superior performance in this regard, with a detection rate of 78.11%. This result is considerably more accurate than those observed for sociodemographic characteristics, biochemical parameters, stent length, SYNTAX score, and NLR, which have been previously investigated and subjected to XGBoost modeling in our study [36,37]. Predicting no-reflow with simple calculable parameters prior to angiography will provide additional information to the operator when performing PCI. As a result, more caution will be exercised when performing PCI in high-risk patient groups.

The findings of this study may encourage the integration of the CALLY index and the XGBoost model into clinical practice. Future research should focus on exploring the relationship between the CALLY index, other biomarkers, and imaging techniques with the no-reflow phenomenon. Recent studies have demonstrated that machine learning models can be successfully integrated into clinical tools, improving diagnostic accuracy and providing real-time support to healthcare professionals [38,39]. However, the development of such tools presents challenges, including user interface design, real-time data processing, and ensuring model interpretability in a way that clinicians can easily understand. Future studies will likely focus on the development of these tools, with the potential for integration into existing clinical systems through web-based dashboards or mobile applications.

Finally, the findings of this study may encourage the integration of the CALLY index and the XGBoost model into clinical practice. It is important for future studies to investigate the association of the CALLY index as well as other biomarkers and imaging modalities with the no-reflow phenomenon. This will be a critical step towards validation in a larger group of patients and generalizability of the results. Furthermore, this study will contribute to the existing body of knowledge regarding multicenter prospective studies.

This study has several limitations. Firstly, the study’s single-center design represents a significant limitation. Furthermore, the applicability of the study findings to other contexts may be constrained. Secondly, the retrospective nature of this study precludes the possibility of determining a definitive long-term prognosis for these patients. Moreover, the absence of long-term follow-up of these patients precludes any meaningful commentary on mortality and cardiac remodeling in this patient group. Further prospective studies with larger patient populations are required to validate the use of the CALLY index in predicting no-reflow and to evaluate its impact on clinical outcomes in a larger cohort. Thirdly, the albumin, lymphocyte, and CRP parameters used to calculate the CALLY index are easily influenced by patients’ medications, comorbidities, and age, making it difficult to attribute this to the no-reflow phenomenon alone.

## 5. Conclusions

In conclusion, the CALLY index and the XGBoost model may demonstrate considerable potential for predicting no-reflow phenomenon in patients with STEMI. The CALLY index may serve as a practical and reliable method for preoperative identification of high-risk patients, utilizing simple biochemical parameters. It is evident that further prospective studies are required to extend these findings by integrating the CALLY index with other diagnostic tools in order to reach definitive conclusions and facilitate more comprehensive patient management.

## Figures and Tables

**Table 1:** Descriptive statistics of clinical and demographic parameters in STEMI patients.

One-Sample Statistics				
	N	Mean	Standart Deviation	Standart Error Mean
Age	1785	58.527	12.1213	0.2869
BMI	1785	26.503	2.0020	0.0474
Diabetes mellitus (%)	1785 (35.1)			
Hypertension (%)	1785 (66.5)			
Smoking (%)	1785 (62.3)			
Glucose	1785	127.841	54.6558	1.2937
Creatinine	1785	0.872	0.2756	0.0065
LDL	1782	108.899	36.6572	0.8684
CRP	1785	1.332	1.5668	0.0371
WBC	1785	10.649	3.2771	0.0776
Hct	1785	42.906	5.6940	0.1348
Number of vessels	1785	1.918	0.7987	0.0189
Stent length	1785	30.299	6.5334	0.1546
SYNTAX score	1785	14.670	5.3117	0.1257
CALLY index	1785	3.8224	8.82368	0.20885
NLR	1785	5.3271	5.10681	0.12091

Abbreviations: BMI; body mass index, DM; diabetes mellitus, HT; hypertension, LDL; low-density lipoprotein cholesterol, CRP; C-reactive protein, WBC; white blood cell count, Hct; hematocrit, NLR; neutrophil-to-lymphocyte ratio.

**Table 2:** One-sample t-test results to predict no-reflow event in STEMI patients.

One-Sample Test						
	Test Value = 0					
	T	df	Sig. (Two-Tailed)	Mean Difference	95% Confidence Interval of Difference	
					Lower	Upper
Age	203.996	1785	0.001	58.5266	57.964	59.089
BMI	559.298	1785	0.001	26.5025	26.410	26.595
Glucose	98.822	1785	0.001	127.8409	125.304	130.378
Creatinine	133.735	1785	0.001	0.8723	0.860	0.885
LDL	125.406	1785	0.001	108.8986	107.195	110.602
CRP	35.905	1785	0.001	1.3316	1.259	1.404
White blood cell	137.294	1785	0.001	10.6493	10.497	10.801
Hematocrit	318.362	1785	0.001	42.9059	42.642	43.170
Number of vessels	101.468	1785	0.001	1.9182	1.881	1.955
Stent length	195.935	1785	0.001	30.2992	29.996	30.602
SYNTAX score	116.685	1785	0.001	14.6700	14.423	14.917
CALLY index	18.302	1785	0.001	3.82238	3.4128	4.2320
NLR	44.059	1785	0.001	5.32709	5.0900	5.5642

Abbreviations: BMI; body mass index, diabetes mellitus, HT; hypertension, LDL; low-density lipoprotein cholesterol, CRP; C-reactive protein, WBC; white blood cell count, Hct; hematocrit, NLR; neutrophil-to-lymphocyte ratio.

## Data Availability

The data presented in this study are available upon request from the corresponding author. The data are not publicly available due to the arrangements made by the Ethics Committee.
